# “Sometimes they used to whisper in our ears”: health care workers’ perceptions of the effects of abortion legalization in Nepal

**DOI:** 10.1186/1471-2458-12-297

**Published:** 2012-04-20

**Authors:** Mahesh Puri, Prabhat Lamichhane, Tabetha Harken, Maya Blum, Cynthia C Harper, Philip D Darney, Jillian T Henderson

**Affiliations:** 1 Center for Research on Environment Health and Population Activities, Kusunti, P.O. Box 9626 Kathmandu, Nepal; 2 Department of Obstetrics and Gynaecology, University of California, Irvine, CA, USA; 3 Bixby Center for Global Reproductive Health, Department of Obstetrics, Gynaecology and Reproductive Sciences, University of California, San Francisco, 3333 California Street, Suite 335, San Francisco, CA 94118, USA

## Abstract

**Background:**

Unsafe abortion has been a significant cause of maternal morbidity and mortality in Nepal. Since legalization in 2002, more than 1,200 providers have been trained and 487 sites have been certified for the provision of safe abortion services. Little is known about health care workers’ views on abortion legalization, such as their perceptions of women seeking abortion and the implications of legalization for abortion-related health care.

**Methods:**

To complement a quantitative study of the health effects of abortion legalization in Nepal, we conducted 35 in-depth interviews with physicians, nurses, counsellors and hospital administrators involved in abortion care and post-abortion complication treatment services at four major government hospitals. Thematic analysis techniques were used to analyze the data.

**Results:**

Overall, participants had positive views of abortion legalization – many believed the severity of abortion complications had declined, contributing to lower maternal mortality and morbidity in the country. A number of participants indicated that the proportion of women obtaining abortion services from approved health facilities was increasing; however, others noted an increase in the number of women using unregulated medicines for abortion, contributing to rising complications. Some providers held negative judgments about abortion patients, including their reasons for abortion. Unmarried women were subject to especially strong negative perceptions. A few of the health workers felt that the law change was encouraging unmarried sexual activity and carelessness around pregnancy prevention and abortion, and that repeat abortion was becoming a problem. Many providers believed that although patients were less fearful than before legalization, they remained hesitant to disclose a history of induced abortion for fear of judgment or mistreatment.

**Conclusions:**

Providers were generally positive about the implications of abortion legalization for the country and for women. A focus on family planning and post-abortion counselling may be welcomed by providers concerned about multiple abortions. Some of the negative judgments of women held by providers could be tempered through values-clarification training, so that women are supported and comfortable sharing their abortion history, improving the quality of post-abortion treatment of complications.

## Background

Unsafe abortion has been a significant cause of maternal mortality and morbidity in Nepal. The high maternal mortality ratio with unsafe abortion as a leading cause of maternal deaths was a strong impetus for abortion legalization [1,2]. The possibility that greater access to safe abortion could save lives and prevent morbidity provided further motivation [3]. Following intensive advocacy and lobbying, and with the support of research evidence, abortion was legalized in 2002 under specific provisions [4]. Under current law, a woman can legally obtain an abortion up to 12 weeks gestation, up to 18 weeks in the case of rape or incest, and at anytime during pregnancy if her life is at risk or the foetus has congenital anomalies [4].

As of Dec 2011, 497,804 women had received abortion services under the law. More than 1200 providers have been trained and 532 sites have been certified as Comprehensive Abortion Care (CAC) sites [5]. Additionally, Nepal introduced medical abortion services in January 2009 on a pilot basis so as to provide an alternative to surgery for women choosing early abortion [6]. The co-packaged product Medabon® (containing 200 mg of mifepristone and 800 microgram of misoprostol manufactured by Sun Pharma, India, for use up to 63 days of gestation) is supplied to accredited comprehensive abortion care/medical abortion clinics. Encouraged by the success of pilot services, the government has allowed scaling up of medical abortion services throughout the country and recently allowed the import, distribution and use of other registered brands of mifepristone and misoprostol tablets through government approved distributors. Besides the sale to women with prescriptions, private pharmacists are allowed to keep stocks of Medabon and supply it to private health facilities on demand although import and over-the-counter sales of any medical abortion pills are illegal in Nepal. However, many other brands of medical abortion pills enter the Nepalese market because of the open border with India. Moreover, clandestine sale of ineffective *Ayurvedic* medicines, based on a traditional healing system, and other indigenous medicines by pharmacists to Nepalese women have been common [7].

Though progress has been made in expanding services, barriers to care still prevent women from accessing safe abortion. Although over 1200 providers have been trained, consistent availability of providers especially in government facilities is a challenge in expanding abortion services [4]. There are an inadequate number of abortion providers to serve the female population of about 14 million, with 49.2% of childbearing age (15–49 years) and low contraceptive prevalence in the country [8]. Limited availability of abortion services in rural areas, insufficient time allocated to patients at abortion sites, long waiting hours, expensive abortion services, and inadequate counselling have all been identified as challenges in implementing abortion services in Nepal [4]. Like other countries, which are in transition to full implementation of abortion law reform, unsafe abortion continues in Nepal [9].

Despite challenges in the delivery of safe abortion services following legalization, there is evidence of a decline in the maternal mortality ratio in Nepal, purportedly due to increased availability of safe abortion care [2,10]. To further the effective implementation of abortion legalization and improve women’s health, the perspectives of health care workers are important because of their contact with women seeking abortion and treatment for abortion-related complications. Their perspectives can help identify areas for improvement in the delivery of safe abortion care. It is also important to understand the attitudes of health workers toward women seeking abortion care, given its often controversial place in society.

Abortion can be characterized as a stigmatized medical service in Nepal and elsewhere in the world [6,11-13]. Stigmatization of abortion patients occurs when women who receive abortion services are ascribed negative attributes such as promiscuity and irresponsibility, are perceived to deviate from feminine ideals, and denunciated for perceived sexual activity outside of social norms [11,12,14]. Abortion is stigmatised in Nepal as it challenges the traditional gender roles of childbearing and is contrary to many teachings of Hindu religion [6,15,16]. Moreover, a qualitative study conducted by Bhandari *et al*. has suggested that abortion can carry connotations of extra-marital relationships, strongly opposed by Nepali socio-religious values [15]. Consequently, due to stigma, women seeking or receiving abortion care may feel less empowered to ask questions, challenge poor treatment, or disclose their clinical history, or they may seek abortion services at higher gestation and visit unsafe providers [11,12,17]. Likewise, pressure to conceal abortion may have cognitive and emotional implications, such as psychological distress, and loss of social and family prestige or honour [13].

There has been little research to date on health care workers perceptions of the impact of abortion legalization in Nepal. Studies conducted elsewhere have explored intentions to provide abortion services, concerns about abortion services, and perceptions of women seeking abortion [18-20]. In a qualitative study conducted with providers in South Africa, the need for a separate clinic for quality abortion services was identified [18]. Similarly, in a qualitative study conducted prior to legalization in Nepal, providers estimated the extent of clandestine unsafe abortion in the country [20]. Survey and opinion polls conducted prior to abortion legalization found that clinicians and paramedics held positive views toward legalizing abortion [4]. Since legalization, no study has explored health care workers’ views on abortion legalization and related issues such as abortion seeking behaviors of women and the implications of legalization for clinical practice. The experiences and perceptions of health care workers post-legalization can offer important information for the planning and improvement of abortion care and implementation of the law. This paper examines health care workers’ views of abortion legalization, and changes that they have observed in their practices.

## Methods

Data for this study come from the qualitative component of a larger ongoing quantitative study of the clinical effects of abortion legalization at four major hospitals in Nepal: two located inside Kathmandu (a teaching hospital and a public hospital) and two outside of Kathmandu (public hospitals), serving rural populations. The qualitative component was designed to complement the quantitative investigation with data on the health care context and record-keeping practices. The hospitals located in Kathmandu are major public tertiary care centers in Nepal and serve a substantial numbers of abortion and post-abortion patients. One hospital outside Kathmandu is a regional level facility for Western Nepal serving women from surrounding districts. Another facility outside Kathmandu lies at the intersection of the eastern and western regions of the country and receives a large number of obstetric and gynaecological cases including abortions.

Thirty-five in-depth interviews were conducted in 2007–2009 with health care workers including physicians, nurses, abortion counsellors, and hospital administrators. Purposive sampling was used to select participants from lists (prepared in consultation with senior hospital staff) of health workers involved in abortion provision and treatment of abortion-related complications, as well as in abortion-related administration.

In-depth interview guidelines were developed in English, translated into Nepali, and pre-tested. Topics included views on abortion legalization, changes in society and work place due to abortion legalization, sex-selective abortion, record-keeping practices, and patient care. Follow-up probes were used with the primary questions to gather more nuanced information. Ethical approval was obtained from the Committee on Human Research at the University of California, San Francisco, the Nepal Health Research Council, and study hospitals. Two authors (Harken and Lamichhane) conducted all interviews in English and Nepali. When conducted in English, a translator was present. On average, each interview lasted about one hour. All individuals approached for interview participated in the study. Participants were fully informed of their option to decline the interview or any question. Verbal consent was obtained from all participants.

A thematic approach was used for data analysis. Interviews were tape-recorded (except one refusal by an interviewee), transcribed, and translated into English (14 interviews were conducted in Nepali). A codebook was developed based on the interview questions and from an initial reading of interview transcripts. Transcripts were then repeatedly read and coded for content. Content codes were grouped into thematic categories. Key quotes that exemplified major themes and concepts are presented in the results. The computer software ATLAS.ti (version Win4.2, Scientific Software development, Berlin) was used for organizing the text, assigning codes and extracting the relevant ones.

## Results

### Profile of participants

Most of the participants were health care providers (14 gynecologists/physicians, 13 staff nurses, and 1 health assistant) and the remaining were administrators (6) and a counselor (1). Participants’ professional experience varied widely (9 months to 37 years) and they had different responsibilities in abortion service provision such as counselling, treatment and record keeping. Of the 35 participants, 26 were female and 9 were male. Most participants provided clinical services, including abortions and treatment of abortion-related complications, and also kept and prepared reports on hospital statistics. Participants worked in the hospitals’ abortion clinics, post-abortion care units, emergency rooms, gynaecological wards, and administrative units. Data were analysed by type of participant to assess whether there were different views or opinions on the major themes, and no major differences were observed.

The key themes emerging from the data analysis were: perceived benefits of abortion legalization, health worker views on abortion patients, concerns about low contraceptive use and repeat abortion after legalization, and abortion disclosure in clinical care before and after legalization. Sex-selective abortion also emerged as a theme during the analysis, and these findings have been published elsewhere [21]. Across these themes, the stigma of abortion was found to intersect with and arise from socio-cultural norms about pre-marital sex, the role of husbands, and gender roles. We therefore discuss these issues where they pertain in the discussion of results.

### Perceived benefits of abortion legalization

Many providers interviewed (23 out of 35) thought legalization improved maternal health and was responsible for a reduction in maternal mortality and morbidity. Participants stated that currently there were fewer post abortion complications compared to the pre-legalization period. Overall the providers expressed positive views of abortion legalization. However, three participants indicated that the abortion complications from rural areas have not declined as in urban areas, believing improvements in access to safe abortion are concentrated in the more populated urban areas.

"*Inside the valley [capital city],] it [complications from abortion] has reduced, but from outside, it’s still the same…because there are more [abortion] centers in the valley, not only the CAC centers, but there are other private centers…Also they are more accessible.*"

"*-Obstetrician/Gynecologist*"

"*Earlier in our hospital some women used to come with severe complications after having an abortion in illegal and unsafe places and from unskilled providers. They used to come with severe complications. Such cases have declined and now we don’t have to deal with them. Women used to come with uterine perforations and at the last stage [close to death], which has declined now.*"

"*-Obstetrician/Gynecologist*"

Participants believed that the types and severity of complications with which patients present at the hospitals had changed. Declines in the number of women presenting with septicaemia, infection, uterine perforation and rupture, septic shock and severe bleeding in the post legalization period were noted by the health care workers. This perception could reflect optimism about the benefits of legalization, and an observable effect of the shift in the safety of abortion.

Participants (14 out of 35) reported that prior to legalization women were more likely to obtain abortions from untrained abortion providers that included auxiliary health workers, traditional birth attendants, and *Ayurvedic* doctors. Consequently, women were more likely to present with complications from the insertion of sticks, sharp objects, catheters, and wooden pieces and the use of unsafe medicines, herbs, honey, cow dung, tree bark, and antiseptics, like Betadine and Dettol. As a result, severe complications such as septicaemia, uterine perforation, septic shock and severe bleeding were common.

"*People who had visited midwives, traditional birth attendants, Vaidyas [Ayurvedic doctors], those who had taken honey*^*a*^*and such other medicines used to come.*"

"*-Nurse*"

"*I have seen women coming with sticks being inserted and they have some kind of a paste, they call “pitix”. I don’t know what it contains but it’s like Fevicol [sticky adhesive used for bonding materials] and they get it from India…..And then there are some herbs.....mostly herbs and sticks and pastes.*"

"*-Obstetrician/Gynecologist*"

Participants suggested that an increasing proportion of women are obtaining safe abortion services from hospitals and certified facilities for abortion care. This rise was attributed by some to women’s greater awareness of and faith in abortion services. Furthermore, they stated that women were seeking treatment at the hospital for abortion-related complications more quickly, rather than waiting until symptoms worsened.

"*It is cheaper here and [women] think that it is safer here … They say that when anything happens you manage, so we come here…They think it’s safe.*"

"*-Obstetrician/Gynecologist*"

"*I think there has been an increase in the awareness level among women and people. So, they immediately come for abortion services [to treat complications]. Maybe for that reason we have not seen many severe cases. When they have bleeding, they come to us.*"

"*-Nurse*"

Nevertheless, a few providers pointed to the need to improve women’s knowledge of the abortion law. In particular, they noted the ongoing issue of women visiting the facilities after they had passed the gestational age limit and, thus, not being able to receive services. They reported that uneducated or poor women who have less information and knowledge about safe services were more likely to present at later dates.

"*[Rich people] know that now there is legal abortion and they ask for the abortion directly— they ask for a doctor. I don’t want this baby, I want an abortion…But poor people do not know [up until] how many weeks they can do the abortion. They come—sometimes they come after 15 weeks, 20 weeks.*"

"*-Health Assistant*"

A few participants (5 out of 35) were concerned that the number of medical abortion related complications was increasing due to the use of ineffective and unregistered medical abortion pills obtained mainly from private pharmacists and chemists and other uncertified health personnel.

"*Now, medical abortion drugs are known to all pharmacists and medical shop keepers. They give drugs to the clients without any assessment. Nowadays, whenever we take the history [of a woman presenting with complications]… everyone will say “after taking the medicine” and when we ask where did you take it, they say from the medical store.*"

"*-Obstetrician/Gynecologist*"

On the other hand one participant stated the need for medical abortion service expansion in the country.

"*Medical abortion service is being piloted in the country…I think it should be promoted to the grass-root levels.*"

"*-Obstetrician/Gynecologist*"

### Views on abortion patients

Participants offered many reasons that women sought abortion: being unmarried, pregnancy as a result of an extramarital relationship, unwanted pregnancy, maternal health problems, sex-selection, contraceptive failure, ashamed to use contraceptives, birth spacing, and for limiting family size. Some participants stated that the reasons women sought abortion were similar both before and after legalization. However, a few health care workers noted a shift in the abortion seeking behaviour of some women and in the composition of patients. They noted that more educated people, those with higher socio-economic status, and unmarried girls used to visit private hospitals or go to India prior to legalization, however, now they are accessing services from government health facilities.

"*The reason for abortion before and now is the same because before legalization some used to have an abortion because of having many children, some because they were unmarried…Though they know about [family planning], many come saying that [the pregnancy] has happened by chance. Some are ignorant about [family planning], some come because their husbands don’t want it, and some feel ashamed to do family planning.*"

"*- Nurse*"

"*Now, rich people also come there. They know that now there is legal abortion and they ask for the abortion directly.*"

"*-Health Assistant*"

Some participants held negative judgments of women’s reason for abortion, suggesting that women seeking abortion held careless attitudes towards ending pregnancy. There was concern among a few (9 out of 35) that society had become more open about sexual relationships and, ‘even the unmarried ones’ were openly coming for abortion service. Negative judgments held by society and themselves toward unmarried women seeking abortion care were also expressed.

"*[Women] are more careless; as they know we have abortion services…They come for an abortion without having a justifiable indication. [They come] even for small indications. People have taken abortion services for granted.*"

"*- Obstetrician/Gynecologist*"

"*The facilities of abortion are being misused…Unmarried women are having abortions frequently as well. I have heard that unmarried women visit the private clinics often.*"

"*- Nurse*"

Abortion among unmarried women had particularly negative connotations, as premarital sex is strongly condemned in Nepalese society. Although socio-cultural values are changing, marriage is predominantly arranged, and the reproductive and marriageable status of young women has social and financial implications to the family. There was concern among some (12 out of 35) health care workers that in the post-legalization period more unmarried and teenaged girls were receiving abortion services. Although legally acceptable, moral and social concerns were raised by the providers on abortion sought by unmarried women. One participant attributed the growing number of abortions among unmarried young women to working in massage parlours and dance restaurants.

"*Many teenagers these days have sexual relationships and they come here and [have an abortion] themselves and do not inform their family, which is a bad thing.*"

"*-Nurse*"

"*There are so many young people, so many dance restaurants, massage parlours. All these things have opened up, you know…So, a kind of prostitution and young people seeking jobs…And then, you know, the incidence of unmarried mothers is on the rise and since abortion is legal now they come.*"

"*-Obstetrician/Gynecologist*"

A few (6 out of 35) participants were unaware of specific provisions of the abortion law that ban sex-selective abortion; allow abortion without husband’s consent, and the legality of abortion regardless of marital status. Misperceptions of the abortion law were, notably, not present among the physicians providing abortion care - two health administrators, one health assistant and three nurses had inaccurate knowledge.

"*They have to come within 12 weeks…and they should be married…and the husband should give the permission.*"

"*- Health Assistant*"

"*It’s illegal for unmarried women to my knowledge…because, you know; the culture does not allow it.*"

"*- Physician (Health Care Administrator)*"

The discontinuity between Nepali culture and the provision of the law permitting women to obtain abortion without a husband’s consent, may pose a barrier to comprehension of the law, even among health-care workers involved in abortion and post-abortion care. Efforts to raise awareness and understanding among health care workers involved in abortion care on the legal provisions could help to correct misunderstandings, but this would not necessarily address their cultural and moral concerns.

### Concerns about contraceptive use and repeat abortion

Several different views were aired by the participants regarding contraceptive use in the legal abortion context. Some participants (10 out of 35) were concerned that less attention was being given to the provision and use of family planning. Two perceived contraceptive use to have declined; while two felt that post-abortion contraceptive use had increased. Overall, participants stressed the need to deliver the message that abortion is not a substitute for family planning, and articulated concerns about contraceptive continuation following abortion.

"*When [women] come here for CAC [abortion] we provide them family planning methods for once [for example] she might take pills for a month but we don’t know if she takes the pills regularly*"

"*- Obstetrician/Gynecologist*"

A few participants (6 out of 35) were concerned about repeat abortion, believing that abortion was taken more lightly in the post legalization period and fearing that it could lead to infertility, pelvic inflammatory disease, and perforation. They described repeat abortions as a negative impact of abortion legalization. However, empathy towards women having multiple abortions was also articulated, as well as the need for efforts to discourage repeat abortion by promoting counselling and family planning.

"*If they do it once or twice, it is fine, but if you go and stay there [at the hospital] you can see the same women coming to have an abortion 4–5 times. However, our objective is not to have her repeat abortion. It also means that she has not received counselling services and she has not been motivated to use family planning or contraception.*"

"*-Obstetrician/Gynecologist*"

### Disclosure of abortion history before and after legalization

Participants indicated that women’s willingness to disclose abortion history was affected by the social context of abortion. Providers found that women were more open in sharing their abortion history post-legalization. This had consequences for documentation practices; a few participants (7 out of 35) mentioned that in the post-legalization period more details were being recorded on induced abortion history including place, person, method and time when the abortion was performed and gestational size at the time of abortion. This practice represents a change from the pre-legalization period where confidential terms were used to denote induced abortion. This change can have important implications for clinical care, as providers reported that women continue to present for complications from unsafe abortions.

"*Now it is easier for us to take their detailed history. [Women] do not fear disclosure or a loss of confidentially. Therefore, it has been easier for us to treat women…They frankly say that they did this and so they came for an examination and treatment. Whereas earlier sometimes we had to take them in a separate room, sometimes they used to whisper in our ears.*"

"*-Obstetrician/Gynecologist*"

Conversely, participants (12 out of 35) spoke of the stigma and need for secrecy that remains in the post-legalization period. Many noted that probing and counselling were still required to take a detailed history on induced abortion in post-abortion care clinics. Furthermore, counselling skills and the provider’s ability to take women into confidence determined the disclosure of abortion history; providers explained to women that an accurate medical history is best for high quality and appropriate care.

"*Even after legalization people do not give their actual history of abortion. After talking for 15–20 minutes they come out with their real history. Most patients do not tell us that.*"

"*-Obstetrician/Gynecologist*"

"*That also depends on how much you counsel [women] because we usually tend to take them into confidence and tell them if you don’t give a correct history you won’t get proper treatment…so usually it is also the skill of the provider on how to get medical history from the patient.*"

"*-Obstetrician/Gynecologist*"

The health workers noted that women’s fear of being scolded by providers for going to the wrong place (non-certified or illegal) was one of the reasons for their hesitation to disclose abortion history after legalization. Furthermore, women attempt to conceal the history of abortions performed beyond the gestational limits allowed for legal abortion. In addition, providers reported that unmarried women tend to conceal their history because they are fearful mistreatment from providers.

"*Unmarried also do it [abortion] and they don’t want to tell about it openly so I think they hide it. The other reason is that they are fearful of being scolded or they are afraid that if they tell that [abortion] then they will not be treated well.*"

"*-Nurse*"

## Discussion

Health care workers held positive views of abortion legalization and marked abortion legalization as an important contributory factor in the overall improvement of maternal health in Nepal. Providers’ beliefs that there has been a decline in the number of maternal deaths, the number and severity of abortion complications, and the number of women visiting with a history of unsafe abortion suggests that legalization is perceived to benefit women’s health. Nevertheless, the results also suggest that there are concerns and ambivalence about some aspects of abortion care after legalization.

A number of participants noted that the higher proportion of women obtaining abortion services from approved health facilities, however, participants also suggested that post-abortion complications were increasing with more use of medical abortion obtained from pharmacies and stores. This view is consistent with findings from a cross-border study in which providers reported treating women with abortion complications after use of medical abortion obtained from medical stores [22]. The stores are visited because of convenience and geographic accessibility; consumers can access drugs, information and advice with relative anonymity; waiting times are short and the services received can be less costly than consulting a physician or other health-care provider in the formal health-care systems.

The expansion of medical abortion has a great potential, but it is important that the drugs be taken appropriately. More accurate, complete information to pharmacists is needed, including encouraging women to seek trained and safe abortion providers before purchasing drugs and to visit certified safe abortion sites. Furthermore, research to characterize the use of different drugs for abortion, and the consequences for women’s health and determine effective strategies to engage private chemists and pharmacists to promote the correct use of safe medical abortion pills is needed.

Similar to a study conducted in South Africa after abortion legalization, providers raised concern on repeat abortion and contraception [18]. They were particularly concerned about women relying on abortion rather than contraception and that unmarried girls were obtaining abortions. The fact that Nepal has high unmet need for family planning and smaller families are desired may necessitate a greater reliance on abortion [2]. Contraception alone may not be sufficient, as shown in a seven-country study where corresponding increases in abortion incidence and contraceptive use were attributed to growing need for fertility regulation [23]. Likewise, the apprehension shown by providers that there was an increase in sexual activity among unmarried girls and people in extramarital relationships can be seen as a part of broader social phenomenon. As pointed out by Whitaker, abortion availability interacts with other social phenomenon such as economic liberalization, urbanization, changing socio-cultural norms, and corresponding changes in sexual practices and attitude towards family life [24].

More active educational campaigns may be needed in Nepal to counteract misperceptions of the law and to foster understanding of the need for abortion. Notably, there were no marked differences in views by the type of provider, except for differences in correct knowledge of the abortion law. Non-physician health care workers and a physician administrator indicated that abortion was not legal for unmarried women or without a husband’s permission, along with other inaccuracies. Educational efforts might begin with those working in health care settings providing abortion and post-abortion care.

Our results suggest that post-legalization, abortion patients continue to be hesitant to share their abortion experiences with providers. Evidence has shown that women limit or alter their history regarding sensitive matters including abortion behavior [25-27]. A study conducted in five different countries among women who had terminated pregnancy revealed that in both restrictive and liberal law settings abortion was viewed as a stigmatized practice [27]. This stigmatization has been found to be connected to influence individuals’ disclosure decisions and behaviors [13,28,29], and likely contributes to non-disclosure of abortion history among women seeking medical care for complications from induced abortion. It also represents a barrier to the therapeutic relationship between the patient and clinician, and fears of judgment could limit women’s willingness to request contraception, contributing to additional unintended pregnancies.

Incorrect knowledge of the abortion law provisions among health workers will pose challenges for compliance with the abortion law while also providing high-quality, comprehensive abortion care. Some participants expressed judgments of women seeking abortion for reasons they believed were not justified. Beliefs that abortion is not allowed, or particularly disapproved of for unmarried women or without a husband’s permission, further underscore the role of stigma in the context of abortion care. Nepal is largely a patriarchal and Hindu society, and women’s expected role of bearing many children is challenged by abortion. Indeed, Bhandari et al, in their qualitative work in Nepal and Bangladesh, characterize abortion-seeking as a process characterised by stigma and shame [15]. Similarly, a qualitative study from Bangladesh revealed that women seeking abortion in difficult circumstances such as unmarried or in an advanced stage of pregnancy experienced unsupportive treatment from providers [30].

One of the potential strategies that can be used to counteract negative views of providers towards abortion patients is value-clarification training. These workshops can be useful in helping providers to maintain balance between their personal belief and professional responsibilities [17] and could be integrated as pre-service or in-service training. Treating abortion as a regular medical service and normalizing abortion in public discourse could be other potential strategies to reduce the stigma surrounding abortion care [12]. De-stigmatizing abortion among health care providers is important for improving women’s health care and further reducing maternal mortality. Additionally, further research is needed to understand the issue of abortion stigma and its impact on women and society.

Although our study results provide new insights into the health care workers perception of abortion legalization, the study has limitations. The health care workers interviewed were not representative of all areas of Nepal despite inclusion of providers from both hill and southern plains (*Terai*) regions, and the number of hospitals and health care professionals interviewed was relatively small and did not include staff from private hospitals and uncertified clinics. Finally, the providers we interviewed – who work at government-certified sites – may be more likely to report strict adherence to legal practices even if their actual practices at times diverge.

## Conclusions

While recognizing the improvements in women’s health achieved through abortion legalization in Nepal, our results indicate ways to further improve abortion access and care quality. Providers were generally positive about the implications of legalization; however, some were concerned about continued unsafe abortion practices. Private pharmacists and chemists may increasingly provide information and medication for women seeking abortion, indicating a need for further research with these health workers.

Health care workers indicated a need to focus on family planning service delivery, including more resources and training for post-abortion contraceptive provision to help prevent repeat unintended pregnancy. The stigma of abortion and negative judgments of women seeking abortion expressed by some providers are barriers to high quality abortion, post-abortion, and contraceptive care. Values-clarification workshops could strengthen the support and clinical care women receive when seeking abortion and abortion-related services. Finally, additional research on women’s experiences with providers and abortion and family planning services in Nepal is needed to understand how best to meet their reproductive health needs in the midst of changing social and demographic conditions.

## Endnote

^a^Honey consumed in large amount is perceived to cause abortion.

## Competing interests

The authors declare that they have no competing interests.

## Authors’ contributions

MP and JTH jointly conceived an outline of the manuscript. MP, JTH and PL prepared the first draft of the manuscript. JTH, MP and MB conceptualized and designed the study. PL and TH were involved in data collection. JTH, MB, CCH, PDD and TH reviewed and revised the manuscript for its intellectual content and clarity. All authors read and approved the final content of the manuscript.

## Pre-publication history

The pre-publication history for this paper can be accessed here:

http://www.biomedcentral.com/1471-2458/12/297/prepub
